# Cost-effectiveness of computed tomography lung cancer screening

**DOI:** 10.1038/sj.bjc.6605260

**Published:** 2009-09-08

**Authors:** Jerome M Reich

**Affiliations:** 1 Earl A Chiles Research Institute, Portland, OR, USA

While the assiduity of Castleberry *et al* in compiling and analysing this huge data assemblage is commendable, regrettably, their conclusion that population CT screening is more cost-effective than symptomatic tumour identification at improving lung cancer (LC) outcomes is based on three demonstrably flawed premises:

1. *Survival is a valid metric of LC screening efficacy.* Efficacy denotes a reduction in mortality. Although it is counter-intuitive, increased LC survival has not proven to be a valid surrogate or proxy for increased life expectancy. Using SEER data, Welch *et al* (2000) reported that in 1950–1954 *vs* 1989–1995, 5-year LC survival more than doubled (from 6 to 14%), while the increase in incidence (249%) was exceeded by the increase in mortality (259%). Similarly, 5-year LC survival in the intervention cohorts of the randomised, prospective, Mayo Lung Program and Czech trials of radiographic screening was more than twice that in the controls. Nevertheless, their mortality exceeded that of the controls (Reich, 2002).
2. *Favourable 5-year survival estimates demonstrate the effectiveness of LC screening.* Effectiveness denotes outcomes in community settings. It presupposes efficacy, the maximum reduction in mortality attainable in centers of excellence in which staffs are highly proficient, subjects are pre-screened to exclude those with clinically significant morbidities, and the ‘healthy volunteer effect’ obtains. As these conditions are not uniformly and comprehensively met in community settings, their outcomes will be predictably less favourable. Since efficacy of LC screening has not been demonstrated, estimates of cost-effectiveness are meaningless.
3. *Overdiagnosis is so infrequent that it can be disregarded.* Overdiagnosis denotes the screen identification of LCs that are clinically irrelevant, that is, that would not have become manifest within the individual's lifetime. On the basis of the excess number of LCs identified in the intervention cohorts *vs* controls in the Mayo Lung Project and Czech screening trials, I estimated that the radiographic overdiagnosis exceeded 25% (Reich, 2008). This estimate is conservative, for the computation assumed that all control cases, many of which were screen-identified, were clinically relevant. Owing to its exquisite sensitivity in identifying small, slow-growing cancers, CT screening overdiagnosis will be quite possibly twice this figure (Reich, 2008).

Because of its import and its critical contribution to the controversy surrounding LC screening, the implications of overdiagnosis deserve elaboration. Although some authors have insisted on its non-existence, advancing in support the well-known lethality of clinically identified LC, it is important to acknowledge that screening identifies a phenotypically less aggressive LC population. A belief in its invariable lethality entails the untenable corollary that, however obtained, a diagnosis of LC confers immunity to death from all other causes. The issue therefore is quantity. In considering the much-disputed point about its frequency, the following should be taken into account. (1) The majority of screen-identified cases are slow-growing stage I adenocarcinomas, whose natural history permits lengthy exposure to competing lethal morbidities, which are particularly common among older smokers. (2) Although volunteers were selected for participation in trials on the basis of their high risk for LC combined with their excellent health and ability to undergo resectional thoracic surgery, competing lethal morbidities were a far more frequent cause of death than LC. For example, in the Mayo Lung Project, non-LC deaths (most of them attributed to coronary artery disease) were sevenfold the deaths due to LC. (3) Individuals disputing the existence of a substantial number of overdiagnosed persons point out the high death rate of persons with stage I LC who decline intervention. This assumption incorrectly imputes LC as the cause of death among many persons whose decision, without doubt, reflects their or their physician's recognition of manifest lethal comorbidities. It is a tautological fallacy to ascribe their deaths to previously diagnosed LC and conclude that stage I LC is therefore invariably lethal.

Overdiagnosis has two insidious effects. First, it favourably biases outcome estimates. As overdiagnosed persons, by definition, die of another cause, their LC survival will be 100% with or without therapy. Thus, their contribution to outcome improvement as reflected in LC survival is entirely spurious. Second, overdiagnosed persons experience the psychological harm and the risks and morbidities of invasive diagnostic procedures and resectional surgery with no possible offsetting benefit. Furthermore, owing to the loss of pulmonary reserve, the courses of their smoking-induced cardiopulmonary comorbidities are foreshortened. Brown *et al* (1993), using SEER database figures, reported that the non-cancer relative hazard of death in persons with LC was nearly threefold that in persons with colon or breast cancer.

*Additional considerations*: The cost estimates of population screening are immense. Per 5-year survival, the authors estimate a cost of 100- to 300-thousand dollars. Even if this enhanced survival translated into a reduction in mortality, its justification, considering other health-related obligations and alternative means of reducing LC mortality, would be open to question. More than 90% of the positive tests in CT trials are false positive, that is, the positive predictive value of a positive test is <10%. The emotional and surgical import of false-positive tests merit emphasis: Wilson *et al* (2008), in a CT screening study of 3642 persons, reported that 41% had non-calcified nodules, 95% of which were non-cancerous. Fifty-four subjects underwent thoracic surgery for LC; half as many (28) underwent thoracic surgery for benign disorders to exclude LC.

In summary, the current evidence indicates no benefit and a high likelihood of harm from mass CT LC screening of the at-risk population.
